# Mining the secreted and membrane transcriptome of *Hyalomma dromedarii* ticks for identification of potential protective antigens

**DOI:** 10.1186/s13071-024-06538-5

**Published:** 2024-11-11

**Authors:** Nahla A. Hussein, Asmaa S. El-Shershaby, Shaimaa Abdel-Moez, Amr E. El-Hakim, Yasser E. Shahein

**Affiliations:** Molecular Biology Department, Biotechnology Research Institute, National Research Centre, Cairo, Egypt

**Keywords:** *Hyalomma dromedarii*, Transcriptome, Vaccines, Membrane associated proteins, Secreted proteins, cDNA library

## Abstract

**Background:**

Members belonging to the tick genus *Hyalomma* function as a multi-host reservoir for several pathogens and important parasites infesting large animals, such as camels, goats, cattle and sheep. In Egypt, there is a high risk of pathogen transmission as camels and cattle are imported from Sudan and Ethiopia and shipped to slaughterhouses and animal markets located in populated areas. *Hyalomma dromedarii* ticks are semi-desert vectors and, similar to other members of the genus *Hyalomma*, characterized by long-term feeding. During this process, different physiological, biochemical and immunological interactions occur within both the feeding ticks and their hosts. These biological changes affect the different tick developmental phases. The aim of this study was to explore the transcriptome of mixed messenger RNAs (mRNAs) collected from *H. dromedarii* eggs, larvae, nymphs and fed and unfed adults, using the Gateway cDNA library prepared in pCMV sport6.1 vector

**Methods:**

The clones were sequenced and searched for potential secreted, membrane-associated or transmembrane (SMaT) sequences. The identified SMaT sequences were compared to the National Center for Biotechnology Information (NCBI) non-redundant protein sequence database using Blastx. Annotation and functional classification were achieved by comparison to sequences in the UniProtKB/Swiss-Prot and VectorBase databases and to the publicly available annotated proteomes of six hard tick species (*H. asiaticum*, *Rhipicephalus sanguineus* sensu lato, *Dermacentor silvarum*, *Rhipicephalus microplus*, *Ixodes scapularis* and *Haemaphysalis longicornis*) in addition to the published *H. dromedarii* sialotranscriptome. For the common sequences, we predicted the physicochemical properties, secondary structures and antigenicity of the fragments similar to matched sequences in the UniProtKB/Swiss-Prot database using three different methods.

**Results:**

The quality-trimmed sequences from the cDNA library revealed 319 SMaT transcripts among 1248 sequenced clones. Annotation of the SMaT sequences using the UniProtKB/Swiss-Prot database revealed only 232 non-redundant sequences with at least one match. According to the UniProtKB/Swiss-Prot and Vectorbase databases, the SMaT sequences were either secreted (extracellular) (29 sequences) or cellular (transmembrane and membrane-associated) (203 sequences). These were classified into 10 functional classes: biogenesis (49 sequences), defense (9 sequences), development (36 sequences), signal transduction (28 sequences), transport (15 sequences), protein modification (33 sequences), homeostasis (6 sequences), metabolism (45 sequences) and miscellaneous/uncharacterized (11 sequences). A total of 60 sequences were shared between *H. dromedarii* SMaT, the sialotransciptome and six other hard tick species. The peptide fragments of these sequences that aligned to proteins from the UniProtKB/Swiss-Prot database were predicted to be promising epitopes and mapped to 10 functional classes at different ratios.

**Conclusions:**

Our immuno-informatics analysis identified 60 sequences common among hard tick species and encoded by *H. dromedarii* salivary glands. These annotated SMaT sequences of *H. dromedarii* will pave the way for the identification and discovery of novel potential protective antigens that are either secreted, membrane-associated or transmembrane.

**Graphical abstract:**

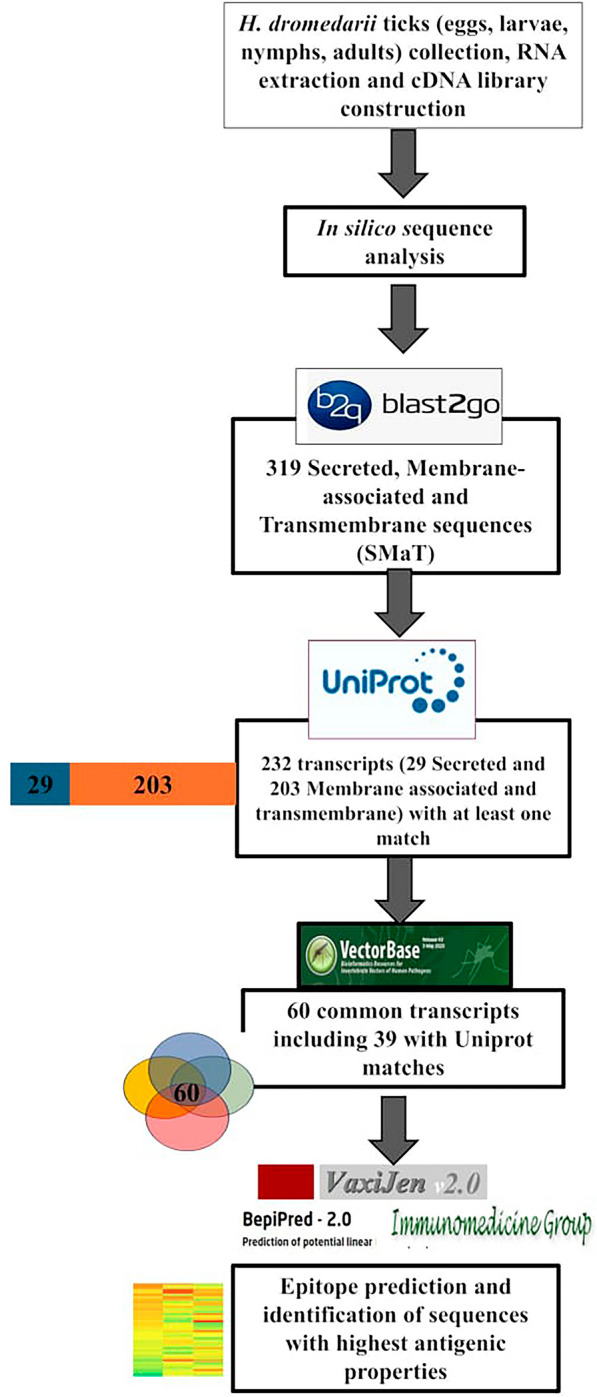

**Supplementary Information:**

The online version contains supplementary material available at 10.1186/s13071-024-06538-5.

## Background

*Hyalomma dromedarii*, a multi-host hard tick similar to other haematophagous arthropods, has major medical significance to both humans and animals. Similar to 44 other species of the Ixodidae family of hard ticks in Egypt, *H. dromedarii* has been reported to infest livestock, both domestic and imported animals for the Egyptian market, including buffaloes, camels, sheep, cattle and goats, as well as dogs and migratory birds crossing the country [1, 2]. Several studies have shown that *Theileria annulata*, *Anaplasma marginale*, *Coxiella burnetii* (Q-fever), *Rickettsia africae* and the Crimean-Congo haemorrhagic fever (CCHF) virus are *H. dromedarii*-borne pathogens that can infect both animals and humans [3–9]. These pathogens cause severe diseases and considerable economic losses.

Traditionally, chemical acaricides have been the most widely and commonly used anti-tick control strategy. However, the use of acaricides has several drawbacks, including harmful effects on the environment, precipitation in milk and meat and, more seriously, the development of acaricide-resistant ticks [10, 11]. As alternatives to the use of these acaricides, numerous biotechnological approaches, including immunization-based methods, have been developed to elicit the host immune response and control tick infestations. These immunological models are based on an understanding of tick biology, especially tick behaviour during blood-feeding. Anti-tick vaccines rely on host immunization with tick proteins found in the mid-gut, salivary glands or eggs or with those associated with Malpighian tubules or cement- like secretions [12–18]. Accordingly, a deeper understanding of tick biology (development, growth, reproduction) may lead to better identification of novel anti-tick vaccines with the potential to efficiently disrupt the tick life-cycle.

Several strategies can be used to identify novel protective antigens that may serve as potential vaccine candidates. Among these, -omics approaches, such as proteomics, transcriptomics and immunomics, have several advantages and have been frequently investigated in the last decade [19]. These provide a generalized overview of ticks proteins/ transcripts that can be upregulated or play essential roles in feeding and reproduction. Examples of such studies include the study of the changes of gene expression during the life-cycle of *Ixodes ricinus* using whole-body RNA [20], the observed clinical signs of *Ornithodoros brasiliensis*-bitten humans using salivary glands RNA [21] and the proteomics of potential epidemiological markers in the sialome of *Hyalomma excavatum* [22]. In addition, the presence of tick-host interacting molecules during blood-feeding and the differential expression of transcripts between male and female ticks in *H. dromedarii* sialome have been reported [23]. In parallel, screening of complementary DNA (cDNA) libraries has aided in the identification of several protective antigens in different ticks species such as *Ixodes scapularis* [24] and *Haemaphysalis longicornis* [25].

However, most studies conducted to date aimed at identifying potential protective antigens have investigated soluble proteins, including cytosolic or secreted extracellular proteins [26]. Membrane-associated and transmembrane proteins, on the other hand, remain poorly investigated, with the notable exception of the prototype Bm86 that is a membrane-anchored protein [18]. This gap could be attributed to the technical challenges encountered when assessing their heterologous expression, such as their low solubility, the need for engineered bacterial expression strains and their tendency to form aggregates [27]. However, cytosolic or secreted extracellular proteins represent promising candidates as they harbor exposed epitopes recognized by the immune system. Their use as antigens may disrupt the functional activities of adhesion, recognition, signaling and regulation [17].

Given this context, there is a potential for using an immuno-informatics approach to predict potential secreted, membrane- associated and transmembrane (SMaT) *H. dromedarii* antigens from the cDNA library. Immuno-informatics plays a crucial role in vaccine development by defining potential antigens from large datasets of proteins and predicting their antigenicity [28]. In this regard, immuno-informatics bypasses the time-consuming steps of in vitro screening and antigen identification.

The study reported here is the first to use an immuno-informatics approach to study the secreted, membrane- associated and transmembrane (SMaT) transcriptome of mixed messenger RNAs (mRNAs) collected from eggs and unfed adults of the hard tick *H. dromedarii*, as well as from *H. dromedarii* at three feeding phases (larvae, nymphs and fed adults). Analysis of the transcripts will give insights into the gene expression profile during growth, development and feeding processes of this tick and will aid in the identification of promising antigens.

## Methods

### Collection and colony establishment of *H. dromedarii* ticks

Engorged females of *H. dromedarii* were collected from naturally infested camels from Toukh City (30°21′11.6″N, 31°11′ 31.5″E), Qalyubia governorate, Egypt. Tick samples were collected at monthly intervals into glass tubes containing strips of filter paper. All collected ticks were examined under a stereomicroscope (model DM 750; Leica Microsystems, Wetzlar, Germany) and identified according to the key of Walker et al. [29]. For oviposition, fully engorged females were placed separately in plastic cups (1 female/cup) that were tightly closed off with a muslin cloth and then incubated at 25 ± 1 °C and 75–80% relative humidity (RH) in a FRIOCELL incubator (MMM Medcenter Einrichtungen GmbH, Planegg, Germany). It is known that fully engorged females begin to oviposit about 5 days after their collection from camels and that females take nearly 1 month to complete oviposition and for larvae to hatch. One week after hatching, the unfed larvae were allowed to feed on healthy rabbits using the capsule technique [30]. After nearly 2 weeks of feeding, fully engorged nymphs were collected from the rabbits and incubated at 25 ± 1 °C and 75–80% RH for 21 days till molting to unfed adults. After 1 week of molting, an equal number of unfed males and females were allowed to feed on healthy rabbits for 10 days to obtain fully fed females from which to collect eggs and ticks at other life stages. This process ensures that all stages used in the library originate from the same mother to avoid polymorphism and diversity.

### Construction of the *H. dromedarii* complementary DNA library

The pCMV-SPORT6.1 vector (Invitrogen, Thermo Fisher Scientific, Waltham, MA, USA) was used to ligate complementary DNA (cDNA) fragments ≥ 800 bp for construction of the *H. dromedarii* cDNA library in pCMV-SPORT6.1 according to the manufacturer’s instructions (Invitrogen, Thermo Fisher Scientific) and applying the basic protocols reported in Sambrook and Russell [31]. RNA was extracted from different tick life stages using TRIzol reagent (Invitrogen, Thermo Fisher Scientific), and the quality of the RNA was examined by electrophoresis, which showed no degradation of the RNA. mRNA was isolated using the Dynabeads Oligo(dT)_25_ mRNA purification kit (Thermo Fisher Scientific). The first-strand cDNA was synthesized using the Invitrogen SuperScript III Reverse Transcriptase kit; The second-strand cDNA was synthesized using the *E. coli* RNase H, *E. coli* DNA polymerase and *E. coli* DNA ligase following the reaction protocol of the Second Strand cDNA Synthesis Kit (A48570; Invitrogen, Thermo Fisher Scientific). Following size selection of the cDNAs by electrophoresis, the cDNAs were directionally cloned into the vector between NotI and EcoRV restriction sites. The EcoRV site is destroyed after cDNA cloning. Electroporation was performed using DH10B-T1R phage-resistant cells (Thermo Fisher Scientific). The primary library was then amplified and stored in suspension medium of 80% SOC and 20% glycerol. The insert size was detected using the NotI and EcoRI restriction enzymes. To carry out sequencing, we used 2× LB broth to dilute an aliquot of the library followed by spreading of each aliquot on LB agar containing 100 μg/ml ampicillin and incubation overnight at 37 °C. Subsequently, colonies were randomly picked into 96-well plates containing 100 μl of 2× LB broth, 8% glycerol and 100 μg/ml ampicillin, and the plates were incubated overnight at 37 °C. After the incubation period, the plates were stored at − 80 °C until subsequent applications. Colonies from these plates were used to inoculate 1-ml cultures of 2× LB broth containing 100 μg/ml ampicillin, and the cultures were incubated for 16 h at 37 °C. Finally, plasmids were purified and sequenced from the 5’ ends using the SP6 primers.

### Identification of potential secreted, membrane- associated or transmembrane (SMaT) sequences

A total of 1248 sequences were analysed using the BLAST2Go annotation tool of OmicsBox Bioinformatics software [32]. We manually searched the output file for all sequences predicted to encode a signal peptide and/or transmembrane helix (based on InterPro classification of protein families and domains which is a part of the BLAST2Go package). Based on the BLAST2Go results, the identified gene ontology (GO terms) were analysed using the GO categorizer (available at https://www.animalgenome.org/bioinfo/tools/countgo [33]) based on root GO annotation followed by Slim2 annotation.

### Sequence annotation and functional prediction

To annotate the *H. dromedarii* SMaT sequences, we used the blastx alignment tool against the UniProtKB/Swiss-Prot database [34, 35] (with default e-value parameters of e-value = 0.05 and maximum hits of 2) and the annotated proteomes of *Hyalomma asiaticum*, *Dermacentor silvarum*, *Rhipicephalus sanguineus* sensu lato (*R. sanguineus* s.l.), *Rhipicephalus microplus*, *Haemaphysalis longicornis* and *Ixodes scapularis* from the VectorBase database release 64 (with a cut-off e-value of 10^–3^) [36, 37]. For sequences common with the six tick species, we conducted an alignment with the UniProtKB/Swiss-Prot database with more stringent blastx parameters (e-value = 10^–3^) to determine the best sequences to be used in subsequent studies for epitope prediction.

### Identification of *H. dromedarii* SMaT salivary proteins

To identify the SMaT sequences encoded by the *H. dromedarii* salivary glands, we used the publicly available sialotranscriptome of *H. dromedarii* [23]. We created a local database of *H. dromedarii *sialotranscriptome sequences and used tblastx (with a cut-off e-value of 10^–3^) for comparison and alignment.

### Physicochemical properties, secondary structure and antigenic prediction:

The physicochemical properties of sequences of interest were predicted using the Multiple Protein Profiler (MPP) 1.0 tool (https://mproteinprofiler.microbiologyandimmunology.dal.ca/) [38] and Phyre2 (http://www.sbg.bio.ic.ac.uk/phyre2/html/page.cgi?id=index) [39].

The potential of the identified *H. dromedarii* SMaT sequences to serve as a potential antigen was predicted using three different tools: Vaxigen- v2 (https://www.ddgpharmfac.net/vaxijen/VaxiJen/VaxiJen.html) [40, 41], BepiPred-2 (http://tools.iedb.org/bcell/) [42] and the tool generated by the Immunomedicine group of University Complutense of Madrid (http://imed.med.ucm.es/Tools/antigenic.pl) [43]. The average score was calculated based on the average of the three prediction scores.

### Graphical representations

Graphical representations were created using GraphPad Prism version 8 (GraphPad Software, San Diego, CA, USA), MS Excel (Microsoft Corp., Redmond, WA, USA), Venn diagrams using the software available at https://bioinformatics.psb.ugent.be/webtools/Venn/ and Heat maps using http://www.heatmapper.ca/ [44].

## Results and discussion

### Construction of *H. dromedarii* cDNA library and sequencing of random clones

**Fig. 1:**
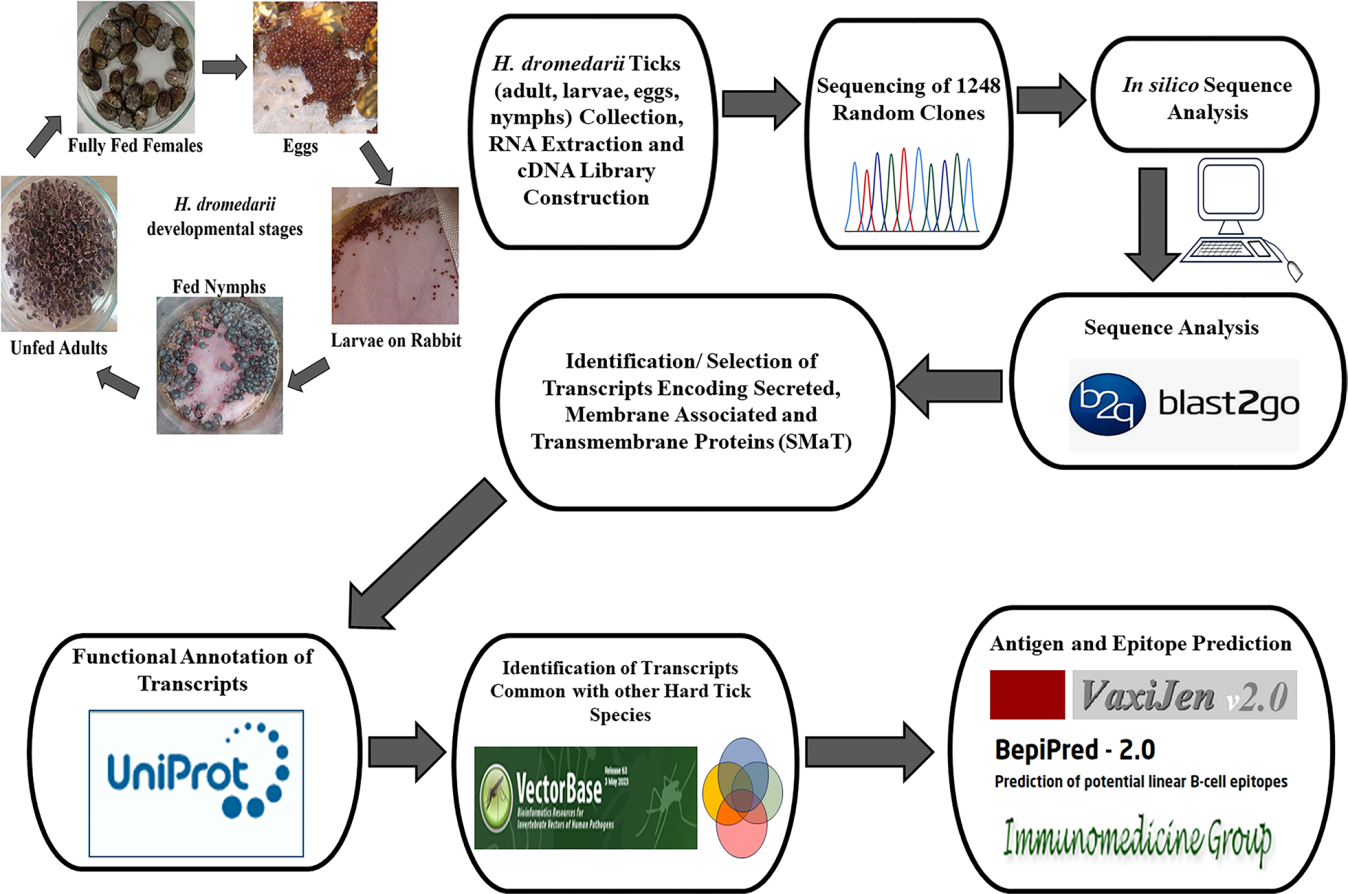
Schematic illustration of the workflow for *Hyalomma dromedarii* transcriptome mining starting from tick colony preparation, collecting the developmental stages, RNA isolation followed by complementary DNA (*cDNA*) library construction and finally sequencing and analysis of transcripts

**Fig. 2:**
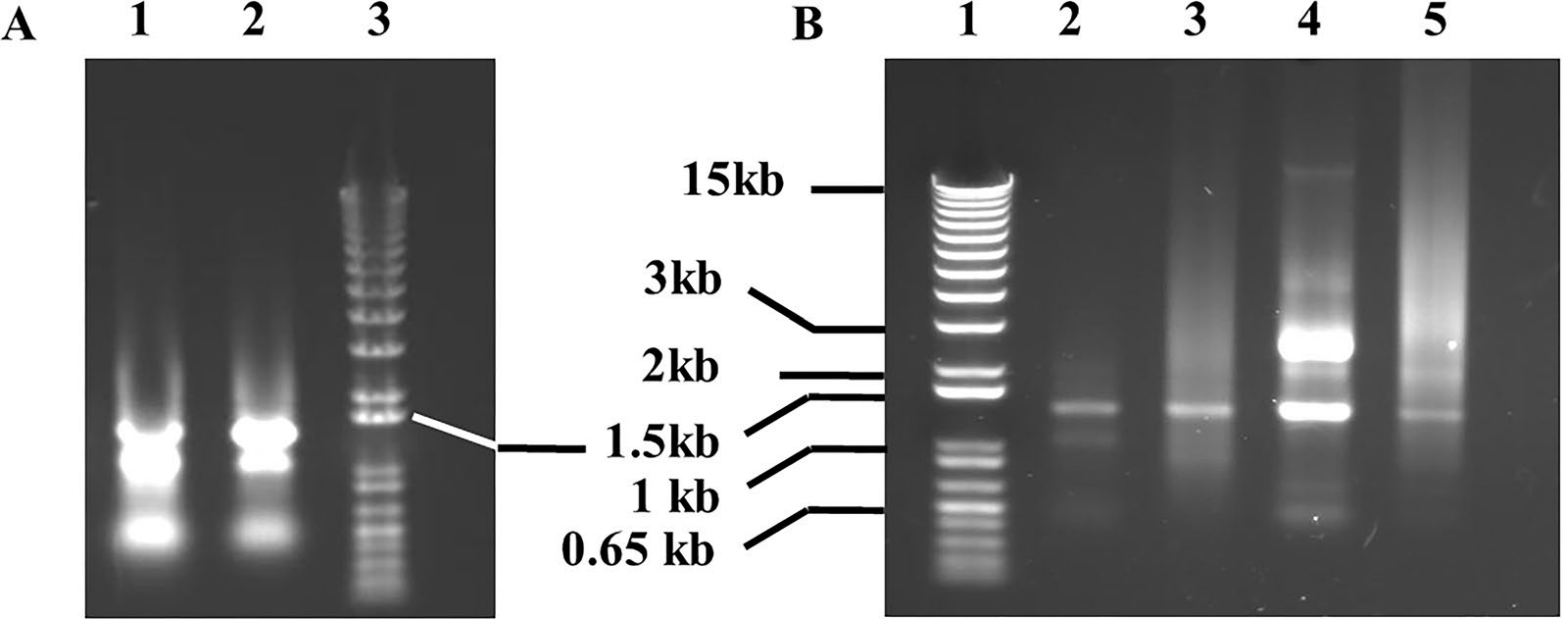
*Hyalomma dromedarii* total RNA and polyA messenger RNA (mRNA). **A** Lanes:* 1*,* 2* tick total RNA,* 3* 1 kb+ DNA ladder. **B** Lanes:* 1* 1 kb+ DNA ladder,* 2* tick total RNA,* 3* tick polyA mRNA,* 4*,* 5* human kidney total RNA and polyA mRNA as a control, respectively

**Fig. 3:**
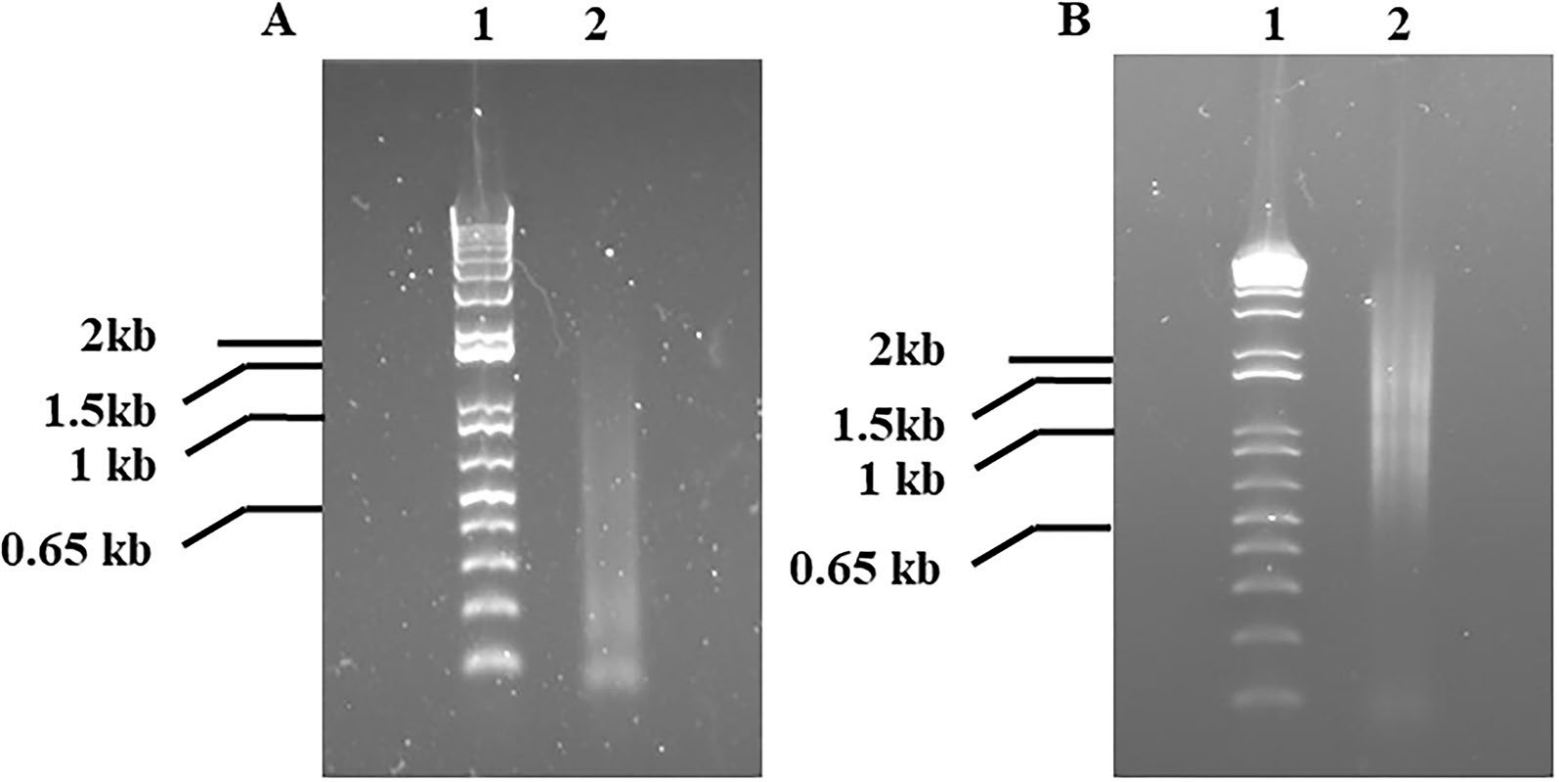
*Hyalomma dromedarii* complementary DNA (cDNA) after Cap-antibody selection. **A** Lanes:* 1* 1 kb+ DNA ladder,* 2* Cap-antibody-selected cDNA from tick. **B** Lanes:* 1* 1 kb+ DNA ladder,* 2* Cap-antibody-selected cDNA from human kidney as a control

In order to gain insights into the *H. dromedarii* transcriptome and identify promising vaccine candidates, we followed the workflow described in Fig. 1. Different life stages of *H. dromedarii* ticks (eggs, larvae, nymphs and adults) were collected after the necessary colony has been established, and aliquots of 100 μg of total RNA from each stage were pooled to further extract the mRNA. The pattern of tick total RNA and ribosomal RNA (rRNA) was similar to the control RNA. The rRNA from ticks (Fig. 2a, and lane 2 in Fig. 2b) showed a pattern of two large bands and one small band, although the sizes of the bands were smaller than those of the control tissue rRNA (Fig. 2b, lane 4). The polyA mRNA from *H. dromedarii* looked similar to that in the human tissue control (Fig. 2b, lane 5), but it contained much more rRNA (Fig. 2b, lane 3) indicating that it bonds to poly-dT oligo. We subsequently synthesized cDNA from poly-dT priming-based reverse transcription, and the size range was much smaller than the expected size, which may reach ≥ 2 kb. Cap-antibody selection trials revealed that the yield was much lower (5- to 10-fold; first-strand cDNA/mRNA) and the size range also smaller (Fig. 3a, lane 2) than those from control tissue. After using fourfold more starting mRNA (2 μg) material for the first-strand cDNA synthesis, Cap-antibody and size selection were conducted and we completed the library following the protocol of the manufacturer using the pCMV-SPORT6.1 (Invitrogen, Thermo Fisher Scientific). The titer of the primary library was 0.39 × 10^6^ cfu/ml and the average size was  approximately 1.2 kb; 96% of the clones contained inserts.

Sequencing of 23 randomly selected primary clones showed that 57% of clones (13 clones) partially or perfectly matched the 16S or 28S rRNAs of *H. dromedarii*, *Dermacentor* sp. or *Dermacentor andersoni*, that 50% of clones were AT-rich (> 95%) RNA sequences that included many of the 16S rRNAs and that 26% of clones from non-AT-rich sequences contained short open reading frame (ORFs). Most of these sequences were novel with no hits (7 clones) using NCBI nucleotide blast analysis, or they showed a partially match to *R. sanguineus* s.l. mitochondrial DNA (GenBank accession: AF081829.1) (1 clone) or were homologues to the *H. dromedarii COI* gene for cytochrome oxidase subunit I (Genebank accession AJ437075.1) (1 clone) and *Haemaphysalis qinghaiensis* clone HqL08 unknown mRNA (Genebank accession EF605264.1) (1 clone). In brief, low yield of cDNA from Cap-antibody selection directed us to enrich the non-ribo mRNA (Fig. 2b, lane 3) and use high-temperature oligo-dT priming for reverse transcription. The library thus produced showed a total of 3.12 × 10^6^ cfu, with 88% of clones containing inserts with an average insert size of 0.8 kb. The library was then amplified, and we reached 5.1 × 10^9^ cfu/ml total titer; 87% of the clones contained inserts. The size of the inserts was detected using NotI and EcoRI restriction enzymes.

Customized classic cDNA libraries and novel next-generation sequencing (NGS) are two methods that are available to identify genetic information of specific RNA. However, both technologies have advantages and disadvantages depending on the target study project or a specified biological, medical or biotechnological application. NGS exhibits higher throughput, more flexibility and greater sensitivity than the sequencing results obtained with the classic cDNA library, and it has the advantage of full exome sequencing and differential gene expression analyses [45]. However, even with the longest reads, in silico assembly is an indispensable step to obtain contigs and scaffolds. Nevertheless, in organisms like *H. dromedarii* where no reference genome/exome is available, in vitro assembly may lead to chimeric sequence generation. Accordingly, we believe a cDNA library is a promising alternative approach if our aim is to identify potential antigenic epitope(s) encoded within single ORFs.

An additional advantage of the classic cDNA library is that it enabled us to obtain stable clones containing fragments of expressed genes from mature mRNA in a vector ready for use in several applications. The applications that can be carried out on our cDNA library may be positive selection of cDNA, direct PCR amplification of the gene, in vitro transcription analysis, screening of plates using a specified probe and/or functional analysis using the eukaryotic CMV promoter.

The novelty of the current work has three aspects. First, the identified transcripts are from different developmental stages of *H. dromedarii*. Second, the identified RNA sequences are in stable constructs ready for subsequent applications. Finally, we focused on the identification of three types of proteins, namely the secreted, membrane- associated or transmembrane proteins. It is important to mention that eggs, larvae, nymphs and adults originated from a stable colony to avoid polymorphism.

In the current study, we faced several challenges during the construction of the *H. dromedarii* library. First, the smaller size range of cDNAs was around 800 nucleotides, which may be attributed to the premature termination of reverse transcription as a result of the secondary structure in rRNAs that dominated in the reaction [46, 47]. Moreover, reactions showed low cDNA yield from Cap-antibody selection, possibly due to the lack/less/different cap structure from tick RNA since most known rRNAs do not contain a regular cap structure. It is worth mentioning that some non-specific RNA (e.g. 16S rRNA) binding to Cap-antibody beads could be carried over, especially when high amounts of beads and mRNA are used . Finally, we could not normalize the library, which may be attributed to the high percentage of AT-rich sequences; instead we performed the non-ribo mRNA enrichment (Fig. 2b, lane 3).

### Identification and preliminary analysis of SMaT sequences within the *H. dromedarii* cDNA library

The sequences obtained from the 1248 clones using the OmicsBox Bioinformatics software [32] were analysed, and the output for all sequences identified as secreted, membrane- associated or transmembrane was searched manually. A total of 448 sequences were identified. All sequences < 250 bp and sequences showing high similarity to the cloning vector pCMV, including the promoter sequences, or sequences encoding the antibiotic resistance marker beta-lactamase were excluded, resulting in 319 sequences used for further analysis, which were submitted to GenBank under accession numbers PP584923–PP585243) (Additional file 1: Table S1 and Additional file 2: Fasta 1).

**Fig. 4:**
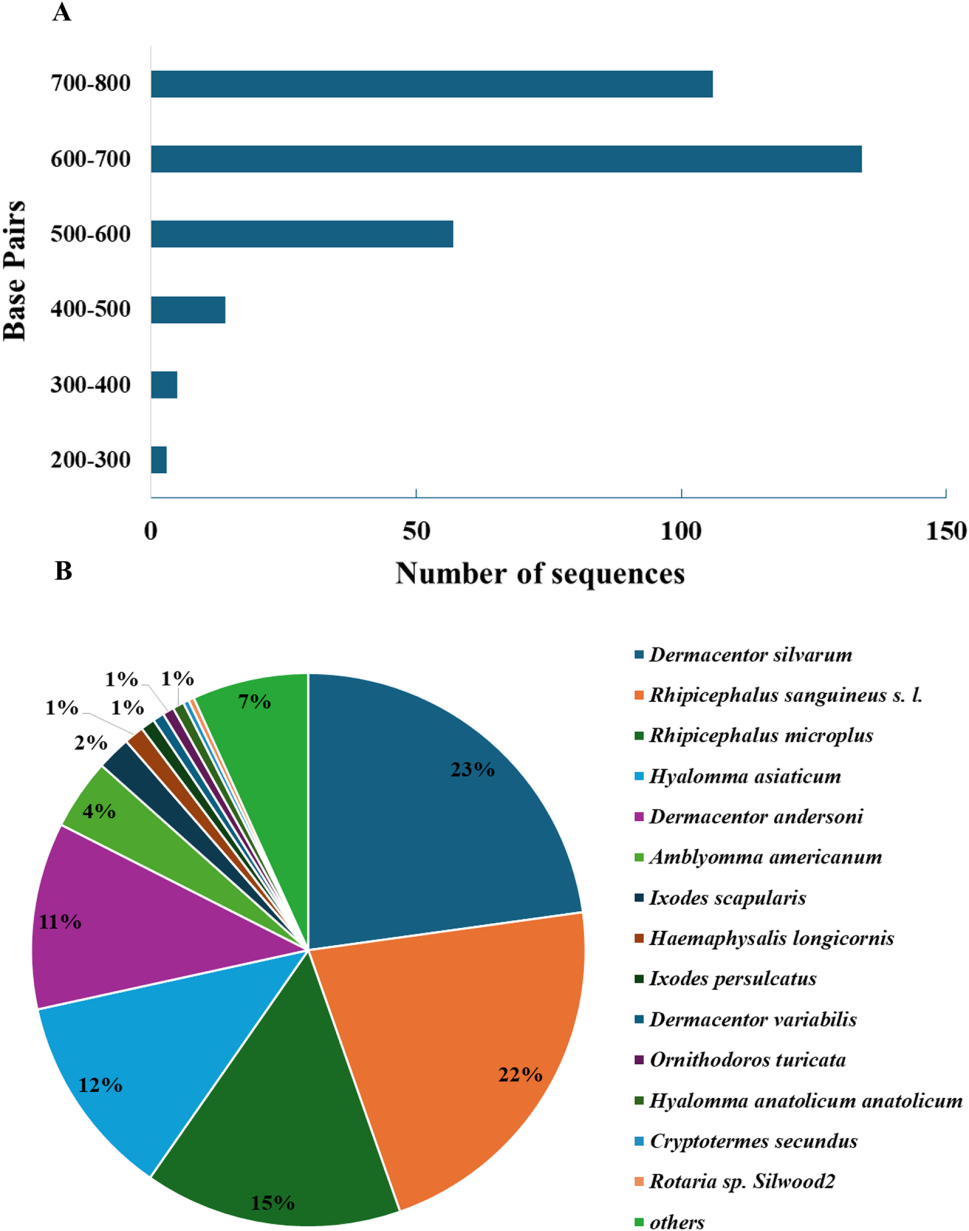
Abundance and length of SMaT-encoding sequences in the *Hyalomma dromedarii* complementary DNA (cDNA) library. **A** Sequence length distribution of the 319 SMaT-encoding sequences used in the analysis. **B** Percentage of *H. dromedarii* SMaT-encoding sequences that had matched hits with other ticks according to alignment using blastx. SMaT, Secreted, membrane-associated or transmembrane

Regarding the length of the *H. dromedarii* SMaT sequences, 106 sequences ranged from 700 to 800 bp, 134 ranged from 600 to 700 bp, 57 ranged from 500 to 600 bp, 14 ranged from 400 to 500 bp, five ranged from 300 to 400 bp and three ranged from 200 to 300 bp (Fig. 4a). Sequence alignment by Blastx against the non-redundant protein sequence database of NCBI using OmicsBox Bioinformatics software [32] revealed that only 126 sequences matched NCBI sequences (including 18 uncharacterized proteins and 58 hypothetical proteins). *Hyalomma dromedarii* SMaT sequences matched hits from several tick species, including *D. silvarum* (23%), *R. sanguineus* s.l. (22%), *R. microplus* (15%)*, H. asiaticum* (12%), *D. andersoni* (11%), *Amblyomma americanum* (4%) and other species (Additional file 1: Table S1; Fig. 4b).

**Fig. 5:**
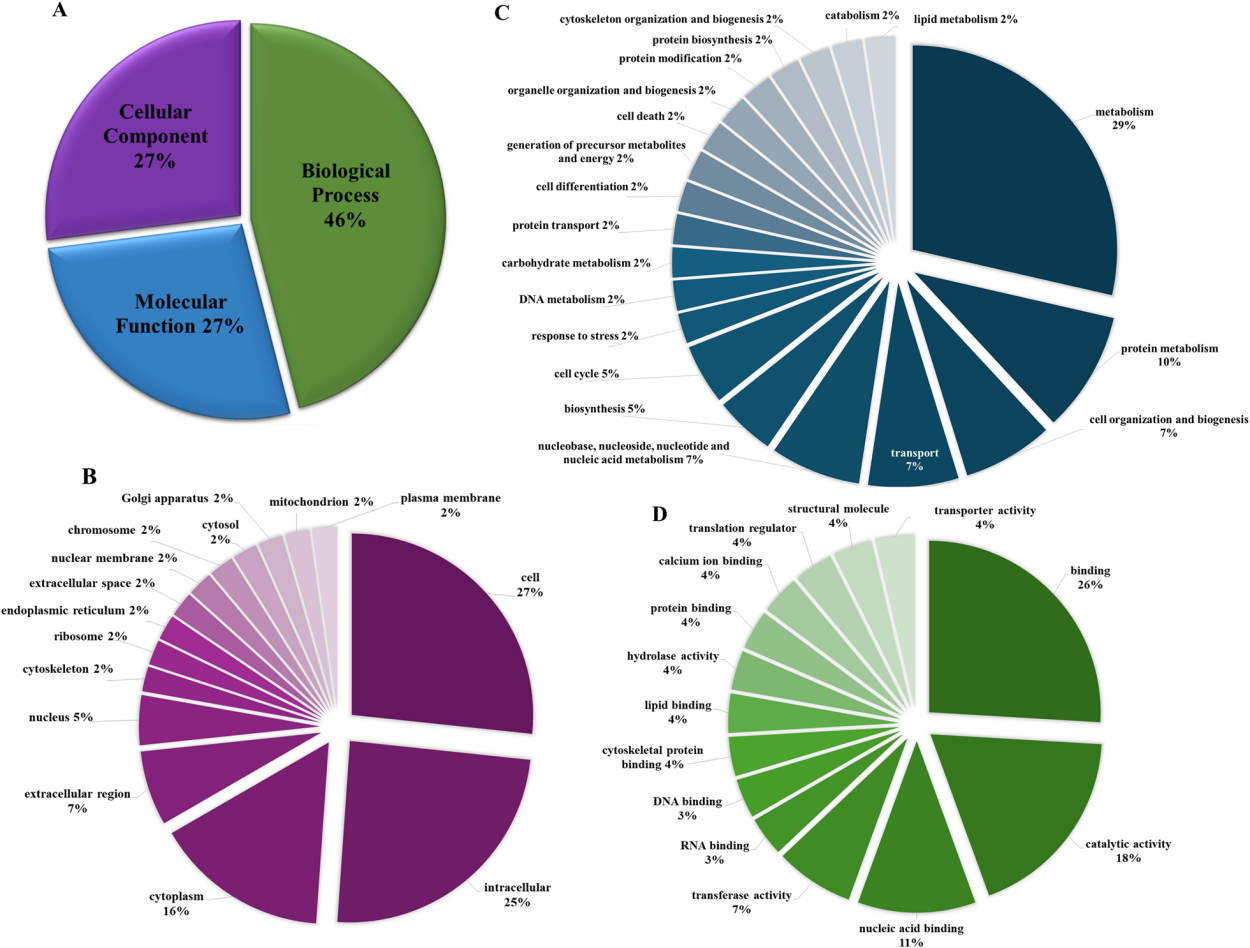
Gene ontology (GO) analysis of *Hyalomma dromedarii* SMaT sequences and their subcategories. **a** Abundance of GO root terms in *H. dromedarii* SMaT sequences identified in this study. **b** Subcategories of GO cellular component terms as detected by the Slim2 framework. **c** subcategories of GO molecular function terms as detected by the Slim2 framework. **d** subcategories of GO biological process terms as detected by the Slim2 framework. SMaT, Secreted, membrane-associated or transmembrane

The 319 SMaT sequences matched 164 GO identities, which fell into the categories of cellular component (27%), molecular function (27%) and biological process (46%), (Fig. 5a**)**. These were further categorized into subcategories according to the Slim2 framework as depicted in Fig. 5B–D.

### Subcellular and functional classification of the *H. dromedarii* SMaT transcriptome

**Fig. 6:**
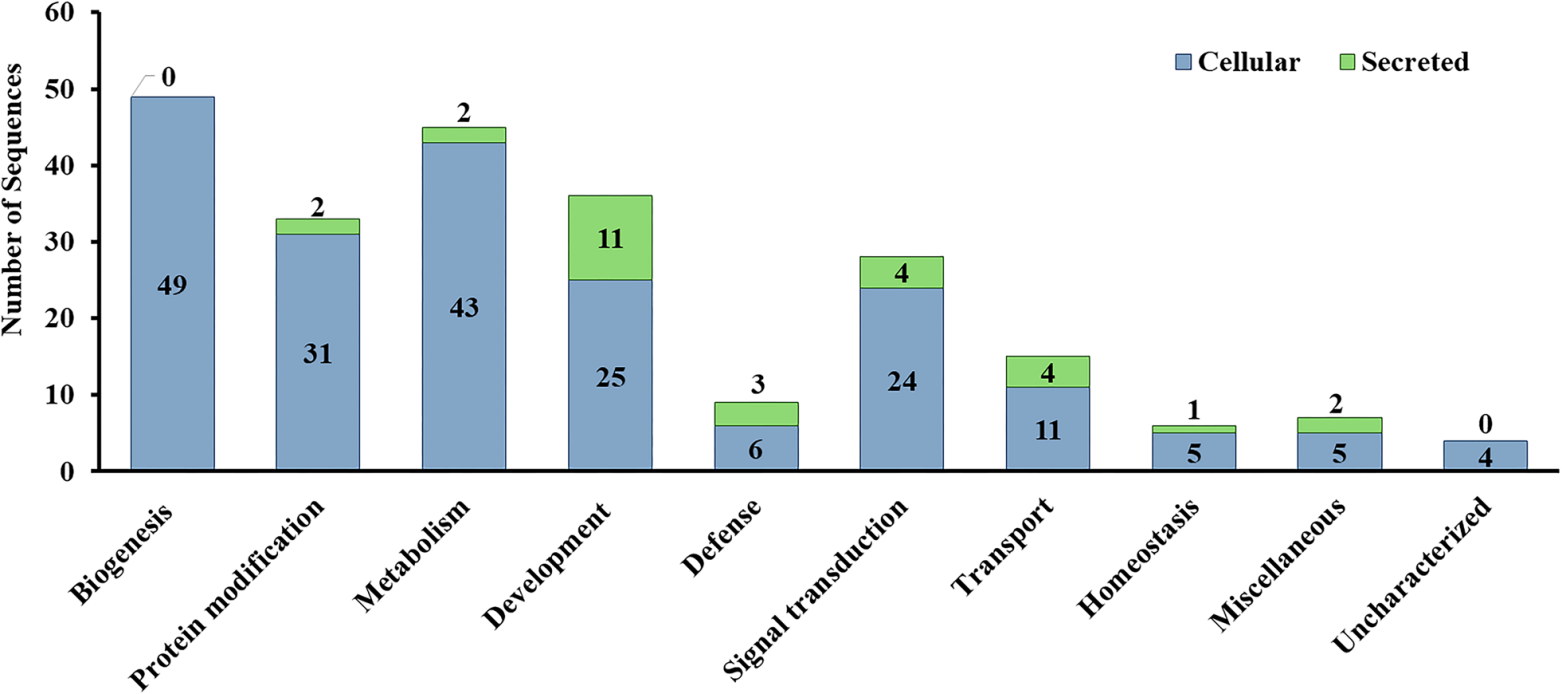
Functional classification of the 232 *Hyalomma dromedarii* SMaT sequences matching UniProtKB/Swiss-Prot entries. SMaT, Secreted, membrane-associated or transmembrane

To classify and predict the function of the identified 319 SMaT sequences, we compared them by blastx to the manually curated database UniProtKB/Swiss-Prot [34, 35], with a maximum of two hits per sequence. Our results revealed that 232 sequences matched hits from UniProtKB/Swiss-Prot (where 48 sequences matched only 1 hit, 184 sequences matched 2 hits (Additional file 3: Table S2) and 87 sequences did not match any hit). We noted the high similarity between sequences P7_H07 and P7_C07 (both matching UniProtKB/Swiss-Prot entry A1KEV7) and between sequences P11_B12 and P13_C08 (both matching UniProtKB/Swiss-Prot entries A4D998 and Q63515), suggesting that each identical pair might originate from the same ORF. Overall, the 232 sequences were predicted to be either secreted (29 sequences) or cellular, including transmembrane and membrane-associated (203 sequences). They were classified into 10 functional classes: biogenesis (including DNA, RNA and protein biogenesis; 49 sequences), protein modification (33 sequences), metabolism (45 sequences), development (including cell division and skeleton formation; 36 sequences), defense (9 sequences), signal transduction (28 sequences), transport (15 sequences), homeostasis (6 sequences) and miscellaneous and uncharacterized (11 sequences) (Fig. 6). The importance and role of some of these functional classes to which the identified *H. dromedarii* SMaT sequences belong are discussed briefly in the following sections.

#### Biogenesis class

This class includes *H. dromedarii* SMaT sequences encoding the synthesis of biomolecules (DNA, RNA and proteins). Most of these sequences are predicted to encode potential ribosomal proteins or ribosomal protein subunits that are involved in ribosome biogenesis and protein translation, such as clones (P1_E07, P2_C02, P3_E08, P4_A03 and P7_E01). In ticks, ribosomal biogenesis is necessary for cell viability, survival and feeding [48]. Ribosomal proteins are involved in many extra-ribosomal functions, including genomic stability, apoptosis, cell proliferation and development [49, 50]. They have also been shown to associate with membrane proteins decorating the cell surface of other parasites, such as *Plasmodium* and *Toxoplasma gondii* [51]. Several studies have highlighted the potential of ticks’ ribosomal proteins as promising antigens if their sequences are conserved among many tick species and are significantly different from the host ribosomal proteins [52, 53]. The authors of one study used *R. microplus* ribosomal protein as a single antigen in bovines and observed reduced tick engorgement, egg-laying and hatching [52]. However, previous studies on *Ha. longicornis* L24 ribosomal protein showed that it is expressed chiefly in ovaries and salivary glands but provides no protection efficiency against tick infestation [54].

#### Defense/immunomodulation

Proteins involved in protective and defense functions play key roles in tick physiology since ticks are exposed to various host blood or skin-borne pathogens, which in turn activates the tick immune system to protect against harmful infections. In this class, we grouped sequences expected to modulate the host immune system or protect ticks against host response.

Among the cellular proteins, we identified tick salivary membrane RING finger protein 150-like (clones P9_D07 and P3_H03). Proteins containing RING domains are characterized by the presence of the zinc binding motif containing seven nonconsecutive cysteines and one histidine. Many of these proteins function as ubiquitin ligase, thus targeting proteins to the proteasome pathway. They contribute also to cell cycle regulation, DNA repair, chromatin remodeling and signal transduction [55, 56]. The sequences of both clones P9_D07 and P3_H03 matched sequences in the annotated proteomes of *R. microplus*, *H. asiaticum* and *Ha. longicornis* (Additional file 4: Table S3). Interestingly, the homologues in *R. microplus* are expected to inhibit the host adaptive immune responses [57].

A third clone, P13_D03, is predicted to encode a polymeric immunoglobulin receptor (PIgR). This receptor functions in anchoring and facilitating the transport of immunoglobulin (Ig)A and IgM across mucosal membrane [58]. PIgRs have not been specifically studied in ticks (to the best of our knowledge) but they may play a role in the neutralization of host antibodies or provision of tick protection against potential pathogens [58].

#### Development

This class includes proteins implicated in ovulation, egg development, cell-cycle progression and cytoskeleton/exoskeleton formation. An interesting example is clone P4_A06, predicted to encode a secreted protein involved in egg yolk development, similar to *R. sanguineus* s.l., vitellogenin-6 protein and UniProtKB/Swiss-Prot entries Q868N5 and Q9U8M0. In ticks, vitellogenin is secreted into the haemolymph and then taken up by the developing oocytes. The main trigger for vitellogenin synthesis in female fat bodies and midgut is engorgement and mating [59]. Vitellins and related proteins have been proven to have a great potential as protective antigens [28]. Interestingly, vitellogenin receptors, which are surface receptors capturing vitellogenin from the haemolymph and promoting their transport and conversion to the active form, were found to present potential targets for tick control [60].

#### Protein modification

Our analysis of *H. dromedarii* SMaT sequences with Swiss-Prot matches revealed 31 sequences involved in protein modification. This class included peptidases, proteases, chaperon-related proteins and some classes of ubiquitin-ligases. These are involved in a variety of physiological processes, including feeding, cellular homeostasis and regulation of cell division [61].

Serine proteases (SP) were among the proteins annotated from the *H. dromedarii* cDNA library. SP are involved in several processes, such as coagulation of blood, inflammation, immune reactions, processing of zymogen, apoptosis, and modeling of matrix [62–64]. For example, clone P11_D05 matched neprilysin-like protein (according to the VectorBase annotation, *R. microplus* proteome, see below), a membrane metalloendopeptidase found to be abundant in the *H. dromedarii* sialotranscriptome [23]. The salivary metalloproteases of ticks are known to play a central role during tick feeding and are involved in several processes, such as coagulation of blood, inflammation, immune reactions, processing of zymogen, apoptosis and remodeling of matrix [62–64]. Interestingly, Neprilysin-like peptidase has been identified as an important antigen eliciting host immune response in *I. ricinus* [65].

Additionally, clone P3_C11 is predicted to encode a thiol protease with dipeptidyl peptidase activity. In addition to its potential role in feeding, it might also play a role in activating other proteases, such as elastases and cathepsins, all of which are involved in feeding, embryogenesis and tissue remodelling [66].

P5_F06 is predicted to encode an ubiquitin conjugating enzyme, which is well known for its role in protein degradation through the proteasome complex. However, accumulating evidence suggests its involvement is immunomodulation both intra-and extra-cellularly [67]. It has also been suggested that ubiquitin-conjugating enzymes are involved in the immunomodulatory effect of *Amblyomma variegatum* saliva on bovine cells [68].

### Comparison of *H. dromedarii *SMaT sequences with proteomes of other hard ticks and *H. dromedarii* salivary gland proteins

**Fig. 7:**
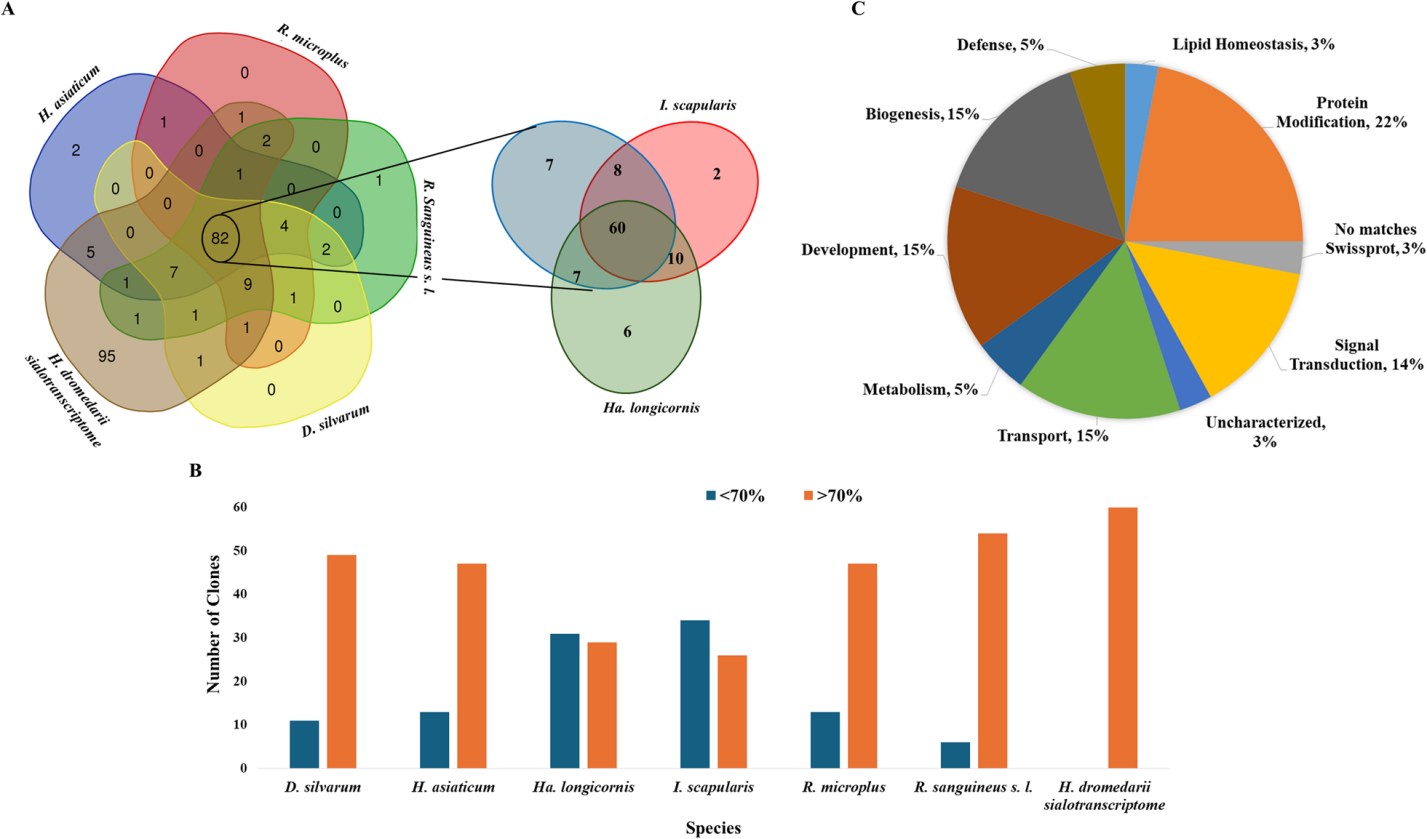
Identification of *Hyalomma dromedarii* SMaT sequences shared and /or similar to those of other tick species. **A** Number of SMaT transcriptome sequences shared between *Hyalomma asiaticum*, *Rhipicephalus sanguineus* s.l., *Dermacentor silvarum*, *Rhipicephalus microplus*, *Hyalomma dromedarii* sialotranscriptome, *Haemaphysalis longicornis* and *Ixodes scapularis*. **B** Number of clones from the 60 common sequences similar to sequences from *D. silvarum, H. asiaticum*, *Ha. longicornis*, *I. scapularis R. microplus, R. sanguineus* s.l*.* and *H. dromedarii* sialotranscriptome with identity lower or higher than 70%. **C** Functional classes of the 60 sequences shared between *H. dromedarii* SMaT and the tick species mentioned above. SMaT, Secreted, membrane-associated or transmembrane

From the results described above, we noted that some *H. dromedarii* SMaT sequences had homologues in the annotated proteomes of other tick species (in particular *R. microplus* and *I. scapularis*). As the aim of this study was to predict potential antigens, we reasoned that transcripts common between different tick species might encode a potential or multi-species protective antigen. Accordingly, to identify *H. dromedarii* SMaT sequences that are common and/or similar to those of other hard tick species, we compared the identified *H. dromedarii* SMaT sequences with the annotated proteomes of the four different species showing the most common hits in the NCBI blastx results (*H. asiaticum*, *R. sanguineus* s.l., *D. silvarum* and *R. microplus*) using the corresponding annotated proteome from VectorBase [36, 37] with an e-value threshold of 10^–3^ (Additional file 4: Table S3). Simultaneously, we compared *H. dromedarii* SMaT sequences to the annotated transcripts of the *H. dromedarii* sialotranscriptome (using tblastn) due to the crucial role of the tick feeding process and the salivary proteins [23]. This comparison revealed 82 common sequences (Fig. 7a; see Additional file 5: List 1 for their IDs). We then compared the 82 common sequences with the annotated proteomes of two other hard tick species, *Ha. longicornis* and *I. scapularis*. All together, these tick species in addition to the *H. dromedarii* sialotranscriptome shared 60 common sequences (the IDs are listed in Additional file 6: List 2). These 60 common sequences were compared to the corresponding homologous sequences from *D. silvarum, H. asiaticum*, *Ha. longicornis*, *I. scapularis R. microplus, R. sanguineus* s. l*.* and *H. dromedarii* sialotranscriptome. For all tick species included in this analysis, the number of *H. dromedarii* SMaT sequences showing > 70% similarity to their homologues was larger than those showing < 70% similarity, with the exception of *I. scapularis* and *Ha. longicornis* (Fig. 7b; Additional file 4: Table S3). The annotation of these 60 common sequences was confirmed by alignment with sequences in the UniProtKB/Swiss-Prot database using more stringent blastx parameters (e-value = 10^–3^) (Additional file 4: Table S3) and were classified into the functional categories shown in Fig. 7c. Among these, 21 sequences (3%) had no matches to those in UniProtKB/Swiss-Prot and were excluded from subsequent analysis, leaving 39 *H. dromedarii* SMaT sequences for further analysis.

### Physicochemical properties, secondary structure and epitope prediction of *H. dromedarii* SMaT potential antigens

To predict the specific amino acid stretches that may have antigenic properties, we extracted the sequence stretches of the 39 SMaT sequences aligning with their UniProtKB/Swiss-Prot homologues (Additional file 7: Fasta 2) and (1) investigated their physicochemical/secondary structure properties and (2) predicted their antigenic potential.

**Fig. 8:**
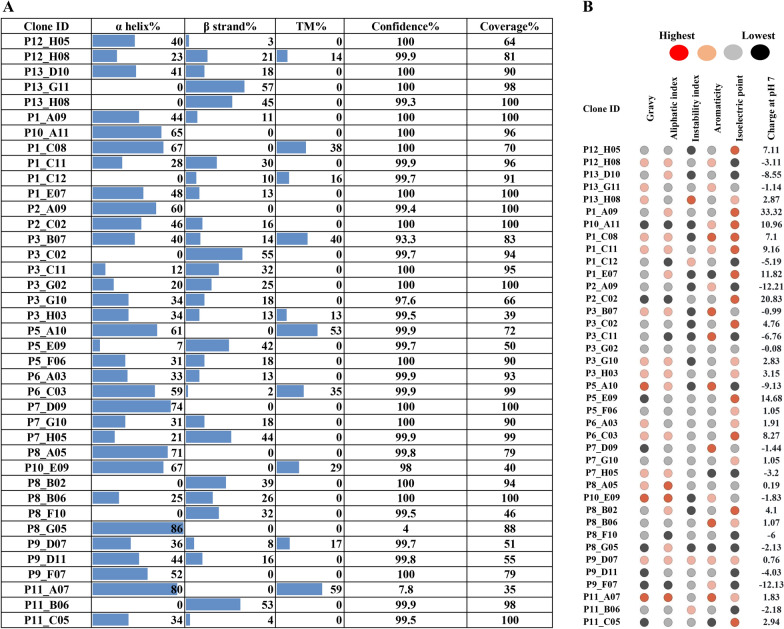
Multiple physicochemical properties of all 39 SMaT sequences. **A** Properties were calculated using the fold recognition server Phyre2. **B** GRAVY index, aliphatic index, instability index, stability, molecular mass, aromaticity, theoretical isoelectric point (pI) and charge at pH 7 were calculated using the MPP 1.0 tool. Gravy, Grand average of hydropathicity (index); MPP, Multiple Protein Profiler; SMaT, secreted, membrane-associated or transmembrane

The MPP 1.0 tool was used to predict the physicochemical properties (Additional file 8: Table S4), including the grand average of hydropathicity (GRAVY) index, aliphatic index, instability index, stability, molecular mass, aromaticity, theoretical isoelectric point (pI) and charge at pH 7 [38]. In addition, the percentage of amino acids forming alpha (α) and beta helices (β), respectively, and the percentage of amino acids constituting transmembrane domains (if present) were calculated using the fold recognition server Phyre2 [39]. Based on the analysis, 17 sequences were considered stable since their dipeptide instability weight values (DIWV) were predicted to be < 40; 22 sequences were predicted to be unstable (Fig. 8a). Nevertheless, the presence of certain dipeptides in a protein structure is one element which contributes to the stability or instability of that protein; other sequence-dependent factors, such as disulphide bonds, type of ligands and their binding and mechanisms of protease recognition, would determine overall protein stability [69].

Regarding the relative volume of a protein occupied by its aliphatic side chains (aliphatic index), the analysis showed that 59% (23 sequences) had high values of > 80 (Fig. 8a). This index is important as a means to control the thermal stability of a protein, where the higher the aliphatic index, the more stable the protein [70]. Contrary to Ikai [70], Panda and Chandra [71] considered cytotoxins and short neurotoxins from *Naja annulifera* and *Naja naja*, with aliphatic indices ranging between 66.5 and 84.33, to be thermostable. In our study, out of 39 SMaT sequences, 10 clones showed similarities to membrane homologues and all of these showed high aliphatic indices, with the exception of P1_C12 clone which showed an aliphatic index of 45.82 (Additional file 8: Table S4). Based on Phyre2 prediction tool (Fig. 8b), this transcript is similar to the low-density lipoprotein receptor (Extracellular domain, Fold library ID c1n7dA) with a confidence of 99.7% and coverage of 91%. Although the sequence of the clone contains 13% serine plus threonine residues and a low content of aliphatic amino acids (24.4%), this predicted receptor binds to metal ions (calcium). It is possible that the presence of large numbers of residues forming hydrogen bonds may contribute to the thermal stability of such proteins regardless of the aliphatic index [39].

The GRAVY values of the 39 SMaT sequences are shown in Additional file 8: Table S4. Of these 39 SMaT sequences, 64.1% (25/39) had negative values, indicating that these proteins are hydrophilic in nature. The most potent hydrophilic peptides were from clones P2_C02 and P9_F07, with GRAVY values of − 1.02 and − 1.05, respectively. Both clones were predicted based on our annotation to play important roles in protein biogenesis or modifications.

**Fig. 9:**
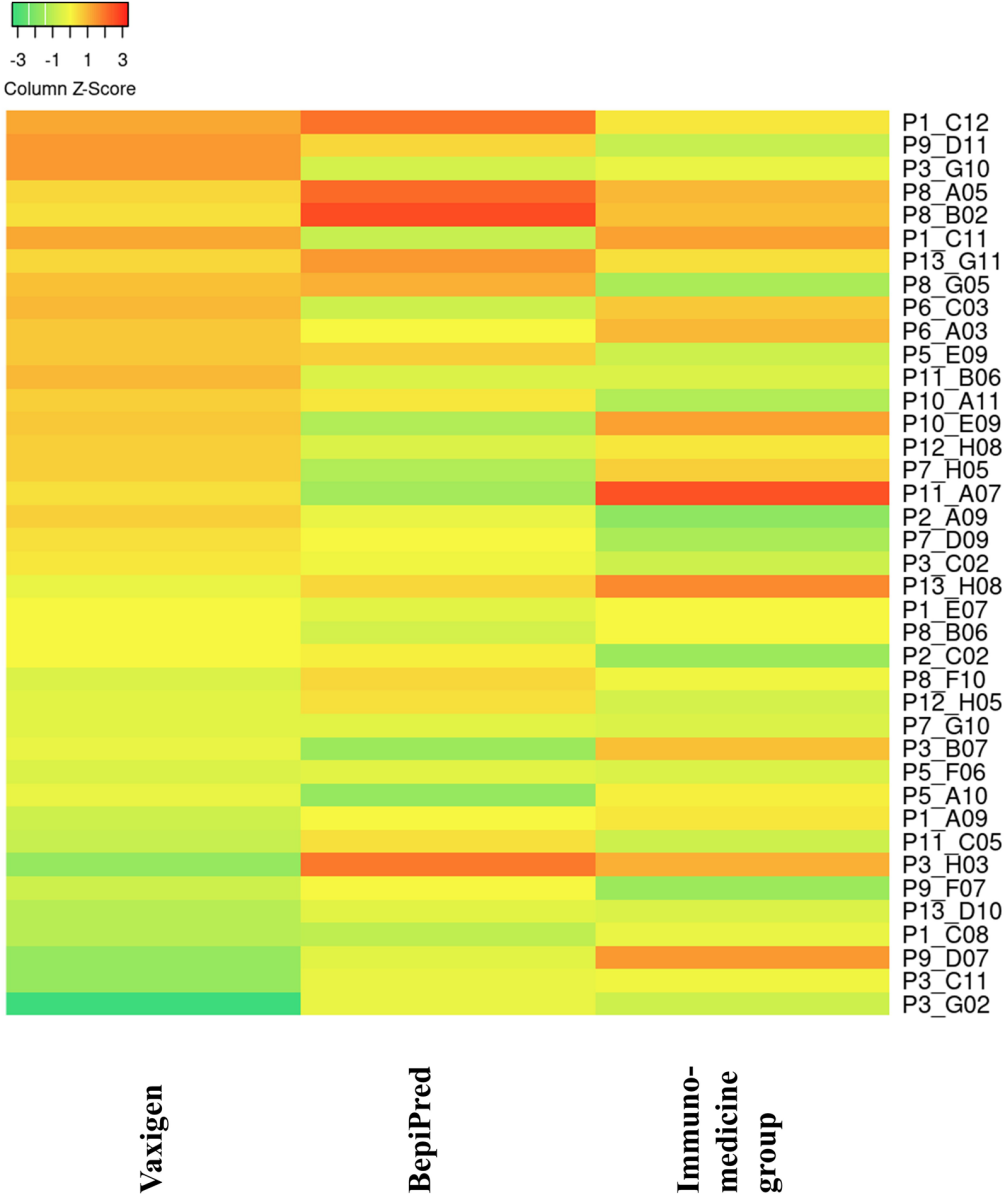
Heat map of antigenic prediction scores of 39 *Hyalomma dromedarii* SMaT sequences using three different prediction tools: Vaxigen (threshold 0.5), Immunomedicine group tool and BepiPred tools [40–43]. Red colouration indicates the highest scores, yellow indicates intermediate scores and green indicates the lowest scores. Prediction was done for the chosen SMaT sequences (common with the tick species mentioned above) using the stretches aligning to those in the Uniprot/Swissprot database. SMaT, Secreted, membrane-associated or transmembrane

Next, using the same sequence stretches aligning to sequences in the Uniprot/Swissprot database, we predicted the antigenicity of 39 *H. dromedarii* SMaT sequences using three prediction tools: Vaxigen (threshold 0.5), Immunomedicine group tool and BepiPred tools [40–43]. The use of three different algorithms for antigenic prediction combines the advantage of relying on the physicochemical properties of the sequences (Vaxigen), on machine-learning algorithms for prediction of B cell epitopes (BepiPred) and on algorithm-based experimentally identified epitopes (immunomedicine group) [40–43]. Based on the prediction results, we created a mean antigenicity score computed from the mean of the three scores (Fig. 9; Additional file 9: Table S5) to select the transcripts with the highest score.

The five sequences displaying highest antigenicity score were (in descending order): P1_C12 (average score 0.805), P9_D11 (average score 0.786), P3_G10 (average score 0.771), P8_A05 (average score 0.77) and P8_B02 (average score 0.767).

P1_C12 is predicted to encode a protein with MAM domain from the low-density lipoprotein (LDL) class. Other than their role in lipid and cholesterol homeostasis, vitellogenin receptors also belong to the LDL-receptor class superfamily. Functional vitellogenin receptors are indispensable for oocyte development and oviposition. Silencing of their encoding gene(s) by RNA interference (RNAi) affects egg-laying capacity [60, 72].

The P9-D11 clone matched a sequence containing the ubiquitin-like (UBX) domain similar to FAS-associated factor 1 (hFAF1)/ UBX domain-containing protein 12)/(UBX domain-containing protein 3A). The UBX domain-containing proteins were found to be phosphorylated salivary proteins participating in protein processing in the endoplasmic reticulum in *Ha. longicornis* [73]. Another model of UBX domain-containing proteins was identified in *I. scapularis* [74]. The results showed that the UBX domain of the p47 protein molecule was capable of binding the tick E3 ubiquitin ligase X-linked inhibitor of apoptosis (XIAP) protein that polyubiquitylates the lysine (K63) of the p47. However, upon infection of *I. scapularis* with *Anaplasma phagocytophilum* and *Borrelia burgdorferi*, the p47 is ubiquitinated via XIAP, which in turn will activate the tick immune deficiency (IMD) pathway to help in tick survival.

The third clone is the P3_G10, which was predicted to code a Pogo transposable element with the Krüppel associated box (KRAB domain). The definite function of the protein is not known, but the KRAB domain is found in Zn-finger (ZnF)-based transcription factors. The domain is considered to be a repressor domain for the transcription process. Accordingly, the KRAB-ZnF proteins are involved in the regulation of several biological processes, such as cell differentiation and proliferation, embryonic development and apoptosis [75, 76].

Clone P8_A05 matched HEAT repeat-containing protein-1 (protein BAP28/U3 small nucleolar RNA-associated protein 10 homolog). Orthologues from different ticks (*D. silvarum*, *I. scapularis*, *R. sanguineus* s.l*.* and *R. microplus*) were annotated and grouped at the Acari level (group number: 15322at6933/https://www.orthodb.org/?query=15322at6933). HEAT-1 protein is poorly characterized to date. Nevertheless, this protein is an important positive player in the biogenesis of ribosomes, and its deficiency will lead to the disruption of ribosome synthesis and consequently p53-independent damage of the DNA [77].

VectorBase resources (release 68, accessed July 2024) showed that *I. scapularis* 5′-AMP-activated protein kinase subunit beta-1 (AMPK subunit beta-1) corresponds to clone P8_B02, is localized in the cytoplasm and plays roles in lipid metabolism, kinase activity and regulation of catalytic processes. However, in ticks, the protein is poorly characterized. In general, the most suggested function for AMPK subunit beta-1 is in male gonad development, production and motility of spermatozoa and the regulation of necessary redox and energy processes needed for fertilization, among other processes [78].

## Conclusions

Construction of the *H. dromedarii* cDNA library revealed the complexity of the *H. dromedarii* transcriptome where the rRNA was dominant during the process of library generation and the mRNA did not contain a regular cap structure as judged by cap-antibody selection. Since eukaryotic cells contain tenfold more rRNA molecules than of other RNA types, these limitations could be overcome in future studies and before direct extraction of mRNA, by depletion of the most abundant rRNA using biotinylated probe oligonucleotides designed to bind ribosomal RNA to enrich the less abundant transcripts. However, mixing RNAs from different life stages of *H. dromedarii* within a library has provided an enhanced deep-sequence ready genetic library to identify and explore novel and unique genes. The sequence data developed herein will pave the way to study gene expression during tick development, create comparative maps with other species and study genetic index of the species. This work provides a start database for *H. dromedarii* SMaT transcripts, including 60 common transcripts between hard tick species and those encoded by *H. dromedarii* salivary glands. The study shows that the secreted transcripts are more immunogenic than membrane-associated or transmembrane proteins, which may steer researchers to novel secreted-in-origin targets. Additional insights on the physicochemical properties of *H. dromedarii* transcripts with UniProtKB/Swiss-Prot matches revealed that their structural composition affects mainly the DIWV, GRAVY and the aliphatic indices. These factors play vital roles in the stability and antigenicity of the transcripts. Nevertheless, the modelling calculations of the transcripts showed 93.3–100% confidence for 94.9% of the studied transcripts (37 transcripts out of the 39 transcripts). More in-depth analyses and experimental studies of these transcripts may help to identify and develop novel targets for vaccine development to minimize economic losses resulting from tick infestation and overcome the different drawbacks of using acaricides and other control methods.

## Supplementary Information

**Additional file 1. Table S1:** H. dromedarii SMaT sequences with corresponding matches from NCBI non redundant protein database.

**Additional file 2. Fasta 1:** Nucleotide sequences of the 319 H. dromedarii SMaT sequences.

**Additional file 3: Table S2.** H. dromedarii SMaT sequences showing matches to UniProtKB/Swiss-Prot database.

**Additional file 4: Table S3.** Comparison of the 319 H. dromedarii SMaT sequences to other hard tick species annotated proteomes from the Vector base database.

**Additional file 5: List 1.** IDs of 82 sequences common between H. dromedarii SMaT, four hard tick species and H. dromedarii sialotranscriptome.

**Additional file 6: List 2.** IDs of 60 sequences common between H. dromedarii SMaT, six hard tick species and H. dromedarii sialotranscriptome.

**Additional file 7: Fasta 2.** Amino acid sequences stretch from the 39 H. dromedarii SMaT sequences aligning to the corresponding UniProtKB/Swiss-Prot matches.

**Additional file 8: Table S4.** Physicochemical properties and secondary structures predictions of amino acid sequences stretch described in Additional file 7.

**Additional file 9: Table S5.** Antigenicity score of the 39 H. dromedarii sequences stretch using three different prediction tools and their average.

## Data Availability

The datasets generated and/or analyzed during the current study are available in the repository of the National Center for Biotechnology Information, under the project PRJEB67593, https://www.ebi.ac.uk/ena/browser/view/PRJEB67593 and GenBank accession numbers PP584923 to PP585243.

## Abbreviations

Ma: Membrane associated (sequences predicted to have a signal peptide and a transmembrane helix)
S: Secreted (sequences predicted to have an N-terminal signal peptide and predicted to have no transmembrane helix.
T: Transmembrane (sequences predicted to have one or more transmembrane domains and no signal peptides)
